# Ætheric Solution of Balsam of Tolu in Chronic Bronchial Catarrh

**Published:** 1853-09

**Authors:** 


### PATHOLOGY AND PRACTICE OF MEDICINE.

*Ætheric Solution of Balsam of Tolu in Chronic Bronchial Catarrh.* By Dr. Roziere.—The author employs, in cases of bronchorrhœa and obstinate cough, also in cases of aphonia, from continued talking and singing, as well as in other chronic affections of the chest, æther. sulphur. 60 grmm.; bals. tolutan. pur. 20 grmm. He puts the mixture into a suitable vessel, and makes the patient inhale the vapor every half hour for two or three minutes.—*Med. Times and Gaz.*

